# Editorial: Endothelial Dysfunction During Inflammation and Alloimmunity

**DOI:** 10.3389/fimmu.2018.02886

**Published:** 2018-12-07

**Authors:** Olaf Penack, Thomas Luft

**Affiliations:** ^1^Hematology, Charité Universitätsmedizin Berlin, Berlin, Germany; ^2^Medizinische Fakultät Heidelberg, Universität Heidelberg, Heidelberg, Germany

**Keywords:** endothelial, dysfunction, alloimmune, transplantation, inflammation

Endothelial cells form the inner lining of blood and lymphatic vessels and they have frequent interactions with immune cells as well as foreign agents (1, 2). Endothelial function is crucially involved in physiologic immunity at different stages including recruitment of leukocytes, angiogenesis, and tissue repair (3–5). The term endothelial dysfunction is widely used to describe the non-physiologic activity of endothelial cells. Endothelial dysfunction plays a role in a variety of human diseases, such as arteriosclerosis, cancer, autoimmunity, and sepsis (6). More recently, a role of endothelial dysfunction during inflammatory diseases and transplantation has been demonstrated (7, 8). This research topic addresses the role of endothelial dysfunction during allo-immune reactions as well as during inflammatory diseases.

The first section deals with endothelial dysfunction and allo-antibody responses in organ transplantation. A mini review article by Cardinal et al. presents the evidence for endothelial injury, its causes and long-term consequences on graft outcomes in kidney transplantation. The authors describe the current view on the involvement of the endothelium on graft rejection including apoptotic pathways, autoantibody production, complement deposition, and microvascular rarefaction. Another concise review article by Cross et al. further deepens our understanding on the role of the endothelium during antibody-mediated rejection of solid organ transplants. The authors picture endothelial cells as “victims” of immune reactions, such as allo-reactive lymphocytes and donor-specific antibodies. Subsequently, they discuss the potential role of endothelial cells as “accomplice” of graft rejection facilitating pro-inflammatory allo-responses by upregulation of MHCII molecules and antigen presentation. The original research article by Morales et al. shows that pre-transplantation levels of anti-beta-2-glycoprotein-I antibodies are useful as a biomarker to predict renal graft loss. The authors prospectively followed a large cohort of 740 renal transplant recipients and found a higher rate of vessel thrombosis and subsequent graft loss in the subgroup of patients with high antibody levels before transplant. This finding implicates that an interventional study on prophylactic anticoagulation in renal transplant recipients with elevated anti-beta-2-glycoprotein-I antibody levels is warranted.

The second section of the research topic addresses new findings on the role of endothelial cells in maintenance of vascular barrier function and potential implications for therapeutically targeting of vascular integrity. Disruption of vascular integrity and increased permeability are associated with inflammation and adverse outcome of organ transplantation. A review article by Rahimi highlights recent advances that have provided new insights into endothelial barrier function and mechanisms involved. He describes that endothelial barrier function and integrity are regulated by highly specialized transmembrane receptors through various distinct mechanisms and signaling cascades. Major pathways acting in destabilization of endothelial barrier function as well as potential implications for therapeutic interventions are explained. The role of the Angiopoietin 2/Tie2 Axis, which regulates vascular leakiness during sepsis (9), is addressed in the review article by Leligdowicz et al. The manuscript summarizes new findings on the mechanisms of Ang/Tie2-mediated regulation of vascular leakiness. Furthermore, the authors explain how the Ang/Tie2 pathway can serve clinically as prognostic biomarker and as therapeutic target using small molecules or monoclonal antibodies. Wang et al. present original research results on the mechanisms of sepsis-related injury of the pulmonary endothelium. In previous work in murine models, the authors found a role of Neutrophils in the damage of pulmonary microvasculature during sepsis (10, 11). In the current manuscript, they demonstrate *in vitro* that human pulmonary endothelial barrier dysfunction under septic conditions is caspase-dependent. Pan-caspase inhibition attenuated septic neutrophil-dependent pulmonary microvascular barrier dysfunction.

The third section contains four articles on different mechanistic aspects of endothelial cells dysfunction during inflammation and how endothelial dysfunction and inflammation are interlinked. Le Gallo et al. commence with a review of the CD95/Fas axis in modulating immune functions via induction of mainly non-apoptotic signaling pathways in endothelial cells. Zhang et al. demonstrate in an original research paper that the endogenous sulfide pathway is involved in endothelial cell inflammation. Cho et al. present novel knowledge on how inflammation and endothelial dysfunction are linked by a process that is termed “endothelial to mesenchymal transition.” Vascular endothelial cells undergo dynamic phenotypic switching during inflammation. Endothelial to mesenchymal transition is a complex biological process in which endothelial cells lose their endothelial characteristics, acquire mesenchymal phenotypes, and express mesenchymal cell markers. This process contributes to vascular dysfunction and is involved in tissue fibrosis, pulmonary arterial hypertension and atherosclerosis. The research topic terminates with a review manuscript of Sedding et al. presenting current knowledge on the role of vasa vasorum angiogenesis in endothelial dysfunction during inflammatory arteriosclerotic plaque formation.

## Author Contributions

All authors listed have made a substantial, direct and intellectual contribution to the work, and approved it for publication.

### Conflict of Interest Statement

The authors declare that the research was conducted in the absence of any commercial or financial relationships that could be construed as a potential conflict of interest.
